# The Protective Mechanism of Antioxidants in Cadmium-Induced Ototoxicity *in Vitro* and *in Vivo*

**DOI:** 10.1289/ehp.10467

**Published:** 2008-02-26

**Authors:** Su-Jin Kim, Hyun-Ja Jeong, Noh-Yil Myung, Min-chol Kim, Jeong-Han Lee, Hong-seob So, Rae-Kil Park, Hyung-Min Kim, Jae-Young Um, Seung-Heon Hong

**Affiliations:** 1 College of Oriental Medicine, Kyung Hee University, Hoegi-Dong, Dongdaemun-Gu, Seoul, Republic of Korea; 2 Vestibulocochlear Research Center of Wonkwang University, Iksan, Jeonbuk, Republic of Korea

**Keywords:** auditory cells, cadmium, caspase-3, caspase-9, ERK, extracellular signal-regulated protein kinase, organ of Corti, reactive oxygen species

## Abstract

**Background:**

Several heavy metals have been shown to have toxic effects on the peripheral and central auditory system. Cadmium (Cd^2+^) is an environmental contaminant showing a variety of adverse effects. Given the current rate of release into the environment, the amount of Cd^2+^ present in the human body and the incidence of Cd^2+^-related diseases are expected to increase.

**Objective:**

The overall aim of this study was to gain further insights into the mechanism of Cd^2+^-induced ototoxicity.

**Methods:**

Cell viability, reactive oxygen species (ROS), mitochondrial membrane potential (MMP), cytochrome c (cyt c), phosphorylated extracellular signal-regulated protein kinase (p-ERK), caspases, morphologic change, and functional changes in HEI-OC1 cells, rat cochlear explants, and mouse cochlea after Cd^2+^ exposure were measured by flow cytometry, immunohistochemical staining, Western blot analysis, and auditory brainstem response (ABR) recording. Mechanisms underlying Cd^2+^ototoxicity were studied using inhibitors of different signaling pathways, caspases, and antioxidants.

**Results:**

Cd^2+^ exposure caused cell death, ROS generation, MMP loss, cyt c release, activation of caspases, ERK activation, apoptosis, and finally auditory threshold shift. Cd^2+^ toxicity interfered with inhibitors of cellular signaling pathways, such as ERK and c-*jun N*-terminal kinase, and with caspase inhibitors, especially inhibitors of caspase-9 and caspase-3. The antioxidants *N*-acetyl-l-cysteine and ebselen showed a significant protective effect on the Cd^2+^ toxicity.

**Conlcusions:**

Cd^2+^ is ototoxic with a complex underlying mechanism. However, ROS generation may be the cause of the toxicity, and application of antioxidants can prevent the toxic effect.

Cadmium (Cd^2+^), a nonessential element widely used in industry, is found as a contaminant in many agricultural products (de Vries et al. 2007). Cd^2+^ contamination of soil and water has raised concern because this metal bioaccumulates in the upper levels of the food chain, including in humans (Satarug et al. 2003). Given its current rate of release into the environment, Cd^2+^ content in the human body is likely to increase in the future (Satarug et al. 2003). Also, Cd^2+^ has genotoxic effects (Bertin and Averbeck 2006) that can lead to a higher incidence of Cd^2+^-related diseases. The liver and kidneys have traditionally been considered the main targets of Cd^2+^ toxicity (Horiguchi et al. 2006; Nishijo et al. 2006); however, recent reports suggest that chronic exposure to low doses of Cd^2+^ may cause neurobehavioral problems in humans and other animals, without any detectable renal damage (Leret et al. 2003; Viaene et al. 2000). These observations emphasize the importance of studying the effects of Cd^2+^ in other organs. Recently, Ozcaglar et al. (2001) reported that hair cells are more sensitive to Cd^2+^ than kidney tubule cells, and that the cochlear component of hearing is more vulnerable to Cd^2+^ toxicity than other parts of the auditory system. However, the ototoxic mechanism of Cd^2+^ on the auditory system is not completely understood.

Apoptosis not only plays an essential role in development and tissue homeostasis but is also involved in a wide range of pathologic conditions (De Martinis et al. 2007; Gatzka and Walsh 2007; Van Heemst et al. 2007). In mammalian cells, there are two major caspase activation pathways: extrinsic and intrinsic. In the extrinsic pathway, binding of the death receptors causes activation of caspase-8, an initiator caspase. In the intrinsic pathway, various forms of cellular stress cause mitochondrial alterations, leading to mitochondrial membrane depolarization (MMP) and the release of cytochrome c (cyt c). In the cytosol, cyt c binds to and activates Apaf-1, which then activates procaspase-9. Active caspase-9 directly cleaves and activates the effector protease, caspase-3. The apoptotic pathway induced by Cd^2+^ is controversial. Some studies strongly suggest that caspases play a key role in Cd^2+^-induced cell death (Kim et al. 2000; Kondoh et al. 2002; Li et al. 2000), whereas other studies suggest that Cd^2+^ induces apoptosis through a caspase-independent pathway (Harstad and Klaassen 2002; Lemarie et al. 2004; Shih et al. 2003). These apparently conflicting data suggest that the pathways of Cd^2+^-induced apoptosis differ according to the cell type or the exposure (cell treatment) conditions.

In mammalian cells, three major mitogen-activated protein kinases (MAPKs) have been defined: the extracellular signal-regulated kinase (ERK) pathway and the stress-activated pathways of the c-*jun N*-terminal kinase (JNK) and the p38 MAPK. These pathways are central components of the intracellular signaling networks that control many aspects of mammalian cellular physiology, including cell proliferation, differentiation, and apoptosis (Roux and Blenis 2004; Wada and Penninger 2004). In general, the ERK signaling cascade is activated by growth factors and is associated with cell survival and proliferation (McCubrey et al. 2007; Puddicombe and Davies 2000). In contrast, p38 and JNK are primarily activated by cellular stress and are often associated with inflammation and apoptosis. However, it has been suggested that these signaling pathways play more complex roles in the regulation of distinct cellular effects. The cellular functions regulated by ERK, p38, or JNK appear to depend on the cell type and stimulus as well as on the duration and strength of the kinase activities. For example, ERK activation is involved in the Cd^2+^-induced G2/M arrest and cell death (Kim et al. 2005). However, the ERK pathway is not involved in the Cd^2+^-induced cytotoxicity of CL3 cells, a lung carcinoma cell line (Chuang and Yang 2001). Overall, these reports suggest that the discrepancies in MAPK activation and Cd^2+^-induced apoptosis might be due to differences in the cell type.

The overall aim of this study was to gain further insights into the mechanism of Cd^2+^-induced toxicity in auditory HEI-OC1 cells as well as *in vivo*. The specific aims were as follows:

- To examine the effect of Cd^2+^ on the generation of reactive oxygenation species (ROS), loss of MMP, release of cyt c, and activation of caspase-3, caspase-8, caspase-9, and ERK in HEI-OC1 cells
- To investigate the Cd^2+^ damage to the arrangements of cochlear hair cells in the basal, middle, and apex turn in the organ of Corti from rats
- To investigate whether Cd^2+^ induces apoptosis in hair cells, Hensen cells, and Claudius cells in the organ of Corti
- To understand the mechanism of Cd^2+^-induced cochlear damage *in vivo* in mice exposed to Cd^2+^ for 30 days
- To investigate the protective effects of *N*-acetyl-l-cysteine (NAC) against Cd^2+^-induced ototoxicity both *in vitro* and *in vivo*.

## Materials and Methods

### Reagents

Fetal bovine serum (FBS), and high-glucose Dulbecco’s modified Eagle’s medium (DMEM) were purchased from GIBCO BRL (Grand Island, NY, USA). Propidium iodide (PI) and 3-(4,5-dimethylthiazol-2-yl)-2,5-diphenyltetrazolium bromide (MTT) were purchased from Sigma Chemical Corporation (St. Louis, MO, USA). Caspase-3, caspase-9, cyt c, phosphorylated-ERK (p-ERK), and total-ERK antibodies were obtained from Santa Cruz Biotechnology (Santa Cruz, CA, USA). The caspase assay kits were supplied by R&D Systems Inc. (Minneapolis, MN, USA).

### Cell culture

The HEI-OC1 cell line was a gift from F. Kalinec (House Ear Institute, Los Angeles, CA, USA). The establishment of an immortal cell line was facilitated using a transgenic mouse, Immortomouse (Charles River Laboratories, Wilmington, MA, USA), which harbors a temperature-sensitive mutant of the SV40 large T-antigen gene under the control of an interferon-gamma–inducible promoter element. The cochlear half-turns from the Immortomice at postnatal day (PND) 7 were cultured on uncoated plastic culture dishes under permissive conditions (33°C) in antibiotic-free DMEM. The cochlear explants were placed at different times under nonpermissive conditions (39°C) and allowed to differentiate for up to 180 days. The explants and cells growing in the tissue regions formerly associated with the organ of Corti were isolated by lifting them with a micropipette after a 2- to 5-min incubation with trypsin-EDTA. A cell line, HEI-OC1, was cloned in the absence of antibiotics, using a limiting dilution method, and characterized. The cells were maintained in DMEM with 10% FBS at 33°C under 5% CO_2_ in air. The HEI-OC1 cells express several molecular markers characteristic of the organ of Corti sensory cells: thyroid hormone, brain-derived neurotrophic factor, calbindin, calmodulin, connexin 26, Math 1, myosin-7a, organ of Corti protein 2, tyrosine kinase receptor B and C, platelet-derived growth factor receptor, and prestin. In addition, the HEI-OC1 cells are extremely sensitive to ototoxic drugs (Kalinec et al. 2003).

### Assessment of hearing function

Auditory brainstem response (ABR), performed under ketamine/xylazine (equivalent to 172.4 mg/kg ketamine and 5.5 mg/kg xylazine) sedation, was used to determine auditory threshold. Threshold was based on the visibility and reproducibility of wave III, according to Bourre et al. (1999). While the animals were under sedation, ABR testing was performed in response to 4-, 8-, 16-, and 32-kHz tone bursts. A computer-based signal-averaging system from Tucker Davis Technologies (Gainesville, FL, USA) was used to collect ABR data. The ABR was recorded by three platinum-iridium needle electrodes placed subdermally over the vertex (positive), the mastoid (negative), and the dorsum area (reference/ground) of the animal. Sound was presented through an Etymotic ER-2 earphone (Elk Grove Village, IL, USA), which was placed directly in the ear canal. The ABR threshold began at 90 dB and decreased in 10-dB steps, and each response was repeated.

### MTT assay

Cells (3 × 10^5^ cells/well) were exposed to various Cd^2+^ concentrations. Cd^2+^ (CdCl_2_, Sigma) was dissolved in phosphate-buffered saline (PBS). Cell viability was determined using an MTT assay. Briefly, after incubation with Cd^2+^, an MTT solution (5 mg/mL in PBS) was added (50 μL/well), and the plates were further incubated for 4 hr at 33°C. The precipitated formazan crystals were dissolved by adding dimethylsulfoxide. The level of absorption was measured using a spectrometer (Molecular Devices, Sunnyvale, CA, USA) at 540 nm.

### Flow cytometry analysis of subG_0_/G_1_

After labeling the cells with PI, the subG_0_/G_1_ cell population distribution was analyzed by flow cytometry. Briefly, cells were harvested after Cd^2+^ treatment, washed with cold PBS, and fixed with 70% ethanol for 60 min. After washing in PBS, the cells were resuspended in 1 mL PBS containing 0.25 mg/mL RNAse A and 0.1 mg/mL PI. The cell samples were incubated in the dark for at least 30 min and analyzed using a FACSCalibur flow cytometer and Cell Quest software (Becton Dickinson, Franklin Lakes, NJ).

### Flow cytometry analysis of MMP and ROS production

The cells (1 × 10^6^/dish) were cultured in the presence or absence of Cd^2+^ and harvested by trypsinization at different time points. The changes in MMP were assessed using the Probe 3,3'-dihexyloxacarbocyanine iodide (DiOC6; Invitrogen, Carlsbad, CA, USA). In this method, the staining intensity decreases when the reagents disrupt the MMP, and quantification of the cells is based on the depolarized mitochondria membranes. Briefly, after trypsinization, cells were washed in PBS and incubated in 50-nM DiOC6 for 30 min. To measure the level of ROS production, cells were loaded with 2.5 μg/mL dihydrorhodamine 123 (DHR123), which was converted to rhodamine fluorescent dye by oxidation. The fluorescence was excited at 450–490 nm, and emission was monitored at 515–565 nm. Fluorescence intensities were analyzed by recording the Fl-1 fluorescence by flow cytometry. The data were collected using a FACscan fluorescence-activated cell scanner with the data acquisition program, QCELL Quest (both from Becton Dickinson).

### Spectrofluorimetric measurement of intracellular ROS generation

Intracellular ROS levels were measured using a fluorescent dye, 2′,7′-dichlorofluorescein diacetate (DCFH-DA). In the presence of an oxidant, DCFH is converted to a highly fluorescent molecule, 2′,7′-dichlorofluorescein (DCF). Cells were cultured overnight on round coverslips, and then cultured in the presence or absence of Cd^2+^ and incubated for 30 min with 5 μM DCFH-DA. The fluorescence intensity was measured using a spectrofluorometer (SHIMADZU Corporation, Kyung Dong, Japan) at an excitation and emission wavelength of 485 nm and 538 nm, respectively.

### Terminal deoxynucleotidyl transferase UTP nick end labeling (TUNEL)

We detected apoptosis using the TUNEL technique (In Situ Cell Death Detection Kit; Roche Applied Science, Mannheim, Germany) according to manufacturer’s instructions. Briefly, cells were fixed with fresh 4% paraformaldehyde in PBS. Cells were washed and incubated with blocking solution (0.3% hydrogen peroxide in PBS) for 15 min and then treated with TUNEL reaction mixture for 60 min at 37°C. The immunolabel was developed with metal-enhanced diamino-benzidine (DAB) solution. For nuclear staining, we used 4′,6-diamidino-2-phenylindole (DAPI) (Dojindo Laboratories, Kumamoto, Japan). Cells were counted (positive vs. total) from five fields per well at 400× using optical microscopy (BX51; Olympus France, Rungis, France).

### Assay of caspase activity

The enzymatic activity of caspase was assayed using a caspase colorimetric assay kit (R&D Systems) according to the manufacturer’s protocol. Briefly, the cells were either untreated or treated with Cd^2+^, then lysed in a lysis buffer. The lysed cells were centrifuged at 14,000 rpm for 5 min. The protein supernatant was incubated with 50 μL reaction buffer and 5 μL caspase substrate at 37°C for 2 hr. The absorbance was measured using a plate reader at a wavelength of 405 nm. Equal amounts of the total protein from each lysate were quantified using a bicinchoninic acid protein quantification kit (Sigma).

### Subcellular fraction for cyt c assay

Cells were harvested, and the pellets were then suspended in 5 volumes buffer A (20 mM HEPES-KOH, pH 7.5, 10 mM KCl, 1.5 mM MgCl_2_, 250 mM sucrose, 1 mM dithiothreitol, 1 mg/mL aprotinin). After homogenizing for 20 strokes, the homogenates were centrifuged at 1,200 rpm for 10 min at 4°C. The supernatant was further centrifuged at 100,000 × *g* for 60 min at 4°C, and the final supernatant was used as the cytosolic fraction. The pellet was further lysed with 0.5 mL buffer A and centrifuged at 12,000 rpm for 10 min at 4°C. The resulting pellet (mitochondrial fraction) was resuspended in buffer A. Aliquots of cytosolic or mitochondrial fractions were used for Western blot analysis of cyt c.

### Western blot analysis

For analysis of the levels of cyt c, caspase-3, caspase -9, and p-ERK, the cells were rinsed with ice-cold PBS and lysed with lysis buffer [1% Triton, 1% Nonidet P-40, 0.1% sodium dodecyl sulfate (SDS), 1% deoxycholate in PBS]. The supernatant was then mixed with an equal volume of a 2× SDS sample buffer, boiled for 5 min, and separated on 10% SDS-PAGE gels. After electrophoresis, the proteins were transferred to nylon membranes by electrophoretic transfer. The membranes were blocked for 2 hr in 5% skim milk, rinsed, incubated overnight at 4°C with the primary antibodies, and washed in PBS/0.5% Tween 20 to remove the excess primary antibodies. The membranes were then incubated for 1 hr with the horse-radish peroxidase–conjugated secondary antibodies (against mouse, goat, or rabbit). After three washes in PBS/0.5% Tween 20, the protein bands were visualized using an enhanced chemiluminescence assay (Amersham, Piscataway, NJ, USA) according to the manufacturer’s instructions.

### Organ of Corti explant culture

The organ-culture procedure was similar to that described previously by Zheng and Gao (1996). Sprague Dawley rats (Dae-Han Experimental Animal Center, Eumsung, South Korea) were sacrificed on PND2, and the cochlea was carefully removed by dissection. The basal, middle, and apex turns of the cochlea were used for further study. The cochlear explants were treated with DMEM containing 10% FBS, Cd^2+^, and 50 μM NAC (Sigma), or a combination, then incubated for 24 hr at 37°C. The cultures were then prepared for histologic analysis. The organ of Corti explants were fixed for 15 min in 2% paraformaldehyde in PBS, rinsed in PBS, incubated in 0.25% Triton X-100 for 2 min, and immersed in tetramethylrhodamine isothiocyanate (TRITC)-labeled phalloidin (1:100 dilution; Sigma) in PBS for 20 min. After rinsing with PBS, the specimens were examined by fluorescence microscopy with the appropriate filters for TRITC (excitation: 510–550 nm; emission: 590 nm).

### Animal experiments

All the experiments were performed using male C57BL/6 mice (Dae-Han Experimental Animal Center) (body weight, 16–18 g), which were housed in stainless steel cages in a temperature-controlled (25°C) room equipped to maintain a 12-hr light:dark cycle. The research was conducted in accordance with the accepted principles for laboratory animal use and care (Institute of Laboratory Animal Resources 1985), and animals were treated humanely and with regard for alleviation of suffering. The animals were distributed randomly into three groups of five animals each and were fed standard chow. Group 1 was used as the control and received untreated water for the study period (30 days). Group 2 received 150 ppm CdCl_2_ in their drinking water, and group 3 received CdCl_2_-treated drinking water and 50 mg/kg NAC by daily intraperitoneal injection.

### Preparation of paraffin sections and immunohistochemistry

One cochlea from each animal was used for immunohistochemitry and the other was used for Western blot analysis. The cochleae were removed, fixed immediately by incubating overnight at 4°C in 4% paraformaldehyde, and decalcified by incubation for 2 weeks in 10% EDTA. The cochleae were then embedded in paraffin, cut into sections (2 μm), and placed on coated slides. Paraffin was removed by incubating in toluene. Sections were then dehydrated in a graded series of alcohol solutions and allowed to dry. The binding sites were saturated by incubating for 20 min with an H_2_O_2_ blocking solution. After washing, the sections were incubated overnight at 4°C with the primary antibodies. After washing with PBS, the sections were incubated with the secondary antibodies for 1 hr. All the antibodies were used at a dilution of 1:200 in buffer. The slides were examined by microscopy (Olympus BX51).

### Statistical analysis

Results shown summarize data from at least three experiments and are presented as the mean ± SE. Statistical evaluation of the results was performed by analysis of variance using a Tukey post hoc test. A *p-*value < 0.05 was considered significant.

## Results

### Cell viability and apoptosis in HEI-OC1 cells

**Figure 1:**
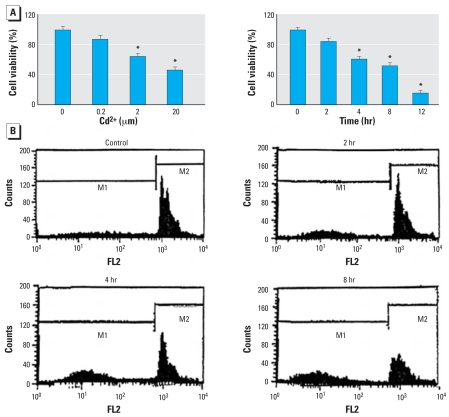
Effect of Cd^2+^ on cell viability and apoptosis in HEI-OC1 cells. Abbreviations: FL2, red fluorescence; M1, first mitosis; M2, second mitosis. (*A*) Viability of cells (mean ± SE) evaluated by MTT colorimetric assay as a function of Cd^2+^ concentration and exposure time. (*B*) Apoptosis in cells as indicated by cell-cycle analysis. *p < 0.05 compared with untreated control cells.

We investigated the effect of Cd^2+^ on the viability of HEI-OC1 cells that were incubated either with different Cd^2+^ concentrations (0.2–20 μM) for 12 hr or with with one Cd^2+^ concentration (20 μM) for varying periods (2–12 hr). Results showed that Cd^2+^ significantly reduced cell viability (measured by the MTT assay) in both a time- and dose-dependent manner (Figure 1A). Cell-cycle analysis was also performed on the HEI-OC1 cells to determine Cd^2+^-induced apoptosis. The data showed that Cd^2+^ induced apoptosis in a time-dependent manner (Figure 1B).

### MMP in HEI-OC1 cells

**Figure 2:**
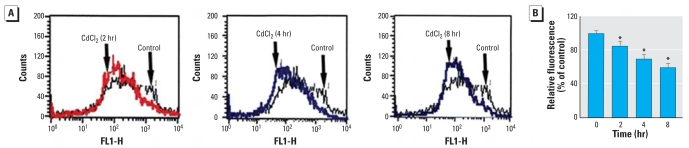
Effect of Cd^2+^ on MMP in HEI-OC1 cells. FL1-H, green fluorescence. (*A*) MMP level measured by flow cytometry using the DiOC6 fluorescent probe; Cd^2+^ incubation (20 μM) resulted in a left shift of the cell distribution, indicating reduced MMP. (*B*) Mean fluorescence intensity (± SE) of traces shown in (*A*). *p < 0.05 compared with untreated control cells.

The loss of mitochondrial membrane integrity is usually one of the first steps in apoptosis triggered by intracellular stress (Delivani and Martin 2006; Javadov and Karmazyn 2007). To determine the effect of Cd^2+^ on the mitochondrial membrane integrity, we incubated cells with Cd^2+^ (20 μM) for varying periods (2, 4, and 8 hr) and measured the level of MMP as an index of mitochondrial membrane integrity. We loaded cells with DiOC6 and measured the fluorescence by flow cytometry. The intensity of DiOC6 fluorescence in Cd^2+^-treated cells was decreased after 2 hr incubation, compared with the control (left shift of the cell distribution). The effect became more pronounced with prolonged incubation time (Figure 2).

### ROS generation in HEI-OC1 cells

**Figure 3:**
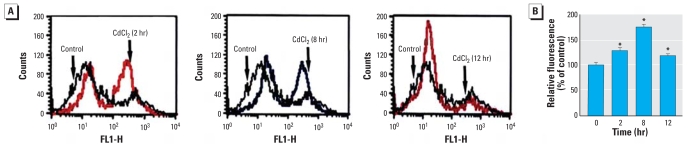
Effect of Cd^2+^ on the level of ROS production in HEI-OC1 cells. FL1-H, green fluorescence. (*A*) Fluorescence measured by flow cytometry using DiOC6 in cells exposed to Cd^2+^ (20 μM) for 2, 8, or 12 hr; the right shift of cell distribution to the high fluorescence area indicates an increase of ROS production. (*B*) ROS levels (mean ± SE) in cells were also measured using the DCFH-DA fluorescent probe and a spectrofluorometer; the relative fluorescence levels were measured and plotted as a function of Cd^2+^ exposure time. *p < 0.05 compared with untreated control cells.

ROS produced in the mitochondria may damage the mitochondrial membrane, leading to apoptosis (Orrenius 2007). We examined the effect of Cd^2+^ exposure on ROS production in cells incubated with Cd^2+^ (20 μM) for varying periods (2, 8, and 12 hr); cells were loaded with DHR123, which converts to the fluorescent molecule rhodamine after intracellular oxidation, and fluorescence was measured by flow cytometry. ROS production increased after Cd^2+^ exposure (right shift of the cell distribution), but the effect became less prominent as exposure time increased (Figure 3A). To confirm the Cd^2+^ effect on ROS generation, the cells were also examined using DCF-DA, which converts to a fluorescent molecule after intracellular oxidation. Figure 3B presents measurements of relative fluorescence levels as a function of Cd^2+^ exposure time.

### cyt c and p-ERK and involvement of different cellular signaling pathways in the Cd^2+^-toxic effect in HEI-COI cells

**Figure 4:**
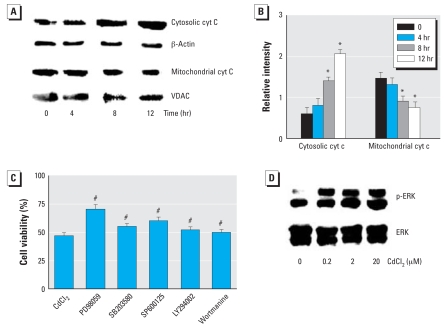
Effect of Cd^2+^ on cyt c and ERK in HEI-COI cells and the involvement of different signaling pathways in Cd^2+^ toxicity. (*A*) Protein extracts assayed for cyt c by Western blot analysis; β-actin was used as internal control in the cytosolic marker, and voltage-dependent anion channel (VDAC) was used as the mitochondrial marker. (*B*) Relative levels of cytosolic and mitochondrial cyt c quantitated by densitometry; the relative intensity of cytosolic cyt c was calculated between the band f cyt c and β-actin, and relative intensity of mitochondrial cyt c was calculated by the ratio between cyt c and VDAC. The change was Cd^2+^ exposure–time dependent. (*C*) Cd^2+^-induced reduction of cell viability, evaluated by MTT colorimetric assay, was partially blocked by preadministration of inhibitors of ERK, JNK, and p38, but the ERK inhibitor was more effective than the others. (*D*) Effect of Cd^2+^ on activation of ERK, showing a dose-dependent activation. Values shown are mean ± SE. *p < 0.05 compared with untreated control cells. ^#^*p* < 0.05 compared with Cd^2+^ alone.

Cyt c is released after mitochondrial membrane permeabilization and induces apoptosis (Garrido et al. 2006). We used Western blot analysis to assay the effect of Cd^2+^ on the release of cyt c in the cytosol. Cd^2+^ treatment increased cyt c levels in the cytosol but reduced cyt c levels in the mitochondria (Figure 4A,B). The relative expression level of cyt c was measured using an image analyzer (Vilber Lourmat FC-26WL, Marne La Vallée, France), and the Cd^2+^-induced changes were exposure-time dependent. In this experiment we also explored involvement of different signaling pathways in the Cd^2+^ toxic effect. The following inhibitors were used: PD98059 (ERK inhibitor, 2 μM), SB203580 (p38 inhibitor, 2 μM), SP600125 (JNK inhibitor, 2 μM), LY294002 (PI3 kinase inhibitor, 0.5 μM), and Wortmanine (PI3 kinase inhibitor, 0.5 μM) (EMD Chemicals Inc., Gibbstown, NJ, USA). The cells were pretreated with the inhibitors and then treated with Cd^2+^. The inhibitors of ERK, JNK, and p38 all showed a more or less protective effect on Cd^2+^ toxicity. However, the ERK inhibitor appeared to be most effective (Figure 4C). Therefore, we examined the Cd^2+^ effect on activation of ERK. As shown in Figure 4D, Cd^2+^ induced the activation of ERK in a dose-dependent manner.

### Caspase activities in HEI-COI cells

**Figure 5:**
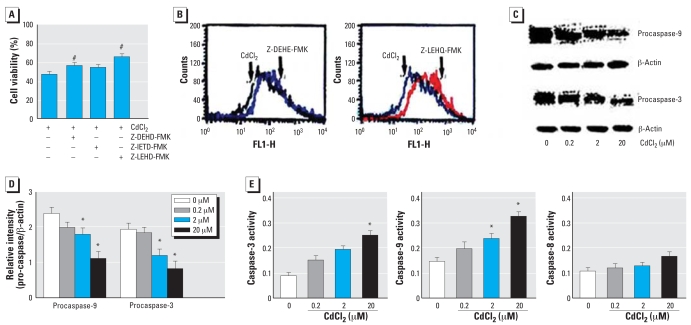
Effect of Cd^2+^ on caspase activities in HEI-COI cells. FL1-H, green fluorescence. (*A*) Cell viability, as evaluated by MTT colorimetric assay, of cells pre-treated with caspase inhibitors (2 μM) for 1 hr and then treated with Cd^2+^ (20 μM) for 8 hr. (*B*) Protective effect of caspase-3 and caspase-9 inhibitors on MMP (right shift), analyzed by flow cytometry using the DiOC6 fluorescent probe. (*C*) Western blot analysis of procaspase-3 and procaspase-9 after Cd^2+^ exposure in a dose-dependent manner. (*D*) Relative levels of the procaspase-3 and procaspase-9 quantitated by densitometry as a function of Cd^2+^ concentration. (*E*) Activities of caspase-3, caspase-8, and caspase-9 as a function of Cd^2+^ concentration, determined using a colorimetric kit. Values shown are mean ± SE. *p < 0.05 compared with untreated control cells. ^#^*p* < 0.05 compared with Cd^2+^ alone.

Proapoptotic stimuli induce mitochondrial membrane permeabilization and promote the release of cyt c in the cytosol, which leads to the activation of proapoptotic factors and the maturation of caspase-3 and caspase-9 (Kroemer and Martin 2005). In the present study, the effect of Cd^2+^ on caspase activities was determined using the following caspase inhibitors: caspase-3 inhibitor (Z-DEHD-FMK), caspase-8 inhibitor (Z-IETD-FMK), and caspase-9 inhibitor (Z-LEHD-FMK) (EMD Chemicals Inc.). The caspase inhibitorsfor caspase-3 and caspase-9, in particular, inhibited Cd^2+^-induced cell death (Figure 5A).The caspase-3 and caspase-9 inhibitors also protected against Cd^2+^-induced loss of MMP (Figure 5B). We performed Western blot analysis to determine the effect of Cd^2+^ on caspase-3 and caspase-9 activities, the inhibitors of which showed significant protection against Cd^2+^ toxicity. Treatment with Cd^2+^ showed reduction of procaspase-3 and procaspase-9, which are inactive forms of caspase-3 and caspase-9, respectively, in a dose-dependent manner (Figure 5C,D). The relative intensity of the procaspase expression level was quantitated by densitometry (Figure 5D). Results showed that Cd^2+^ increased the activity of caspase-3, caspase-8, and caspase-9 in a dose-dependent manner, although the caspase-8 activities did not reach statistical significance (Figure 5E).

### Effect of antioxidants on Cd^2+^-induced apoptosis

**Figure 6:**
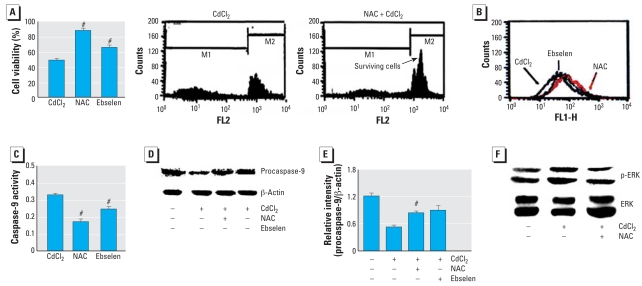
Effect of antioxidants (50 μM NAC or 20 μM ebselen) on the toxic effect of Cd^2+^ (20 μM) in HEI-COI cells. Abbreviations: FL1, green fluorescence; FL2, red fluorescence; M1, first mitosis; M2, second mitosis. (*A*) Cell viability, determined by MTT colorimetric assay and flow cytometry, after exposure to Cd^2+^ (20 μM) or Cd^2+^ plus antioxidant. (*B*) Change in MMP level shown by flow cytometry; note the increase of MMP in cells treated with Cd^2+^ plus antioxidant compared with those treated with Cd^2+^ alone. (*C*) Effect of antioxidants on caspase-9 activation by Cd^2+^ exposure. (*D, E*) Effect of antioxidants on Cd^2+^-induced reduction of procaspase-9 shown by Western blot analysis (*D*) and relative intensity; NAC appears to have more protective effect on Cd^2+^ toxicity than does ebselen. (*F*) Effect of NAC on Cd^2+^-induced ERK activation. Values shown are mean ± SE. #*p* < 0.05 compared with Cd^2+^ alone.

We used NAC and ebselen to determine if antioxidants can regulate Cd^2+^-induced apoptosis. First, an MTT assay and cell-cycle analysis were performed to determine the effect of antioxidants on cell viability. The cells were pretreated with NAC (50 μM) or ebselen (20 μM), then treated with Cd^2+^ (20 μM). NAC and ebselen significantly protected against Cd^2+^-induced cell death (Figure 6A). The antioxidants also inhibited the loss of MMP induced by Cd^2+^ (characterized by a right shift of the cell distribution in Figure 6B). The Cd^2+^-induced caspase-9 activity (Figure 6C) and reduction of procaspase-9 (Figure 6D,E) were blocked by the antioxidants. NAC appeared to have more protective effect than ebselen on Cd^2+^ toxicity. Thus, we focused on the protective effect of NAC. As shown in Figure 6F, Cd^2+^-induced the activation of ERK was blocked by NAC.

### Effect of Cd^2+^ on organ of Corti explants

**Figure 7:**
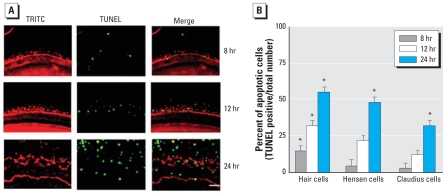
Toxic effect of Cd^2+^ on explants of rat organ of Corti. (*A*) TRITC-conjugated phalloidin (red) and TUNEL (green) staining in organ of Corti explants from rats. Bar = 50 μm. (*B*) Percentage of apoptotic cells (mean ± SE) presented as a function of Cd^2+^ exposure time. Cells were counted from five fields per well (×100) using optical microscopy. *p < 0.05.

The organ of Corti was isolated from rat cochlea on PND2 and treated with Cd^2+^ (10 μM) for varying periods (8, 12, and 24 hr). TRITC-conjugated phalloidin, which binds to F-actin, was used to stain hair cells, Hensen cells, and Claudius cells in the cochlear explant cultures. TUNEL staining (green) was used to detect apoptosis. The Cd^2+^ treatment destroyed the orderly arrangements of the three rows of outer hair cells (OHCs) as well as a single row of inner hair cells (IHCs) and induced apoptosis in the hair cells, Hensen cells, and Claudius cells in a time-dependent manner (Figure 7A). Figure 7B presents the percentage of apoptotic cells as a function of exposure time in different type of cells in the explants.

### The protective effect of NAC against Cd^2+^ toxicity in organ of Corti explants

**Figure 8:**
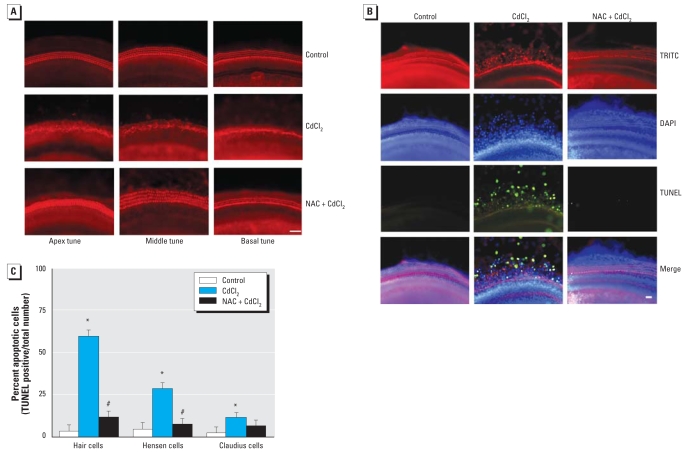
NAC protection against Cd^2+^ damage in sections of cochlear explants from rats. (*A*) Sections of explants from apical, middle, and basal turns of rat cochlea incubated with Cd^2+^ alone or Cd^2+^ + NAC and stained with TRITC-conjugated phalloidin. NAC treatment showed a protective effect against the Cd^2+^ toxic effect. (*B*) Sections of basal turns of cochlea explants stained with TRITC-conjugated phalloidin (red), DAPI (blue), and TUNEL (green) and examined at a magnification of 100×. In (*A*) and (*B*), bar = 50 μm. (*C*) Percentages of TUNEL-positive cells in different groups of cells. *p < 0.05 compared with untreated control explants. ^#^*p* < 0.05 compared with Cd^2+^ alone.

We isolated the organ of Corti in the apical, middle, and basal turns in the rat on PND2 and treated the explants with Cd^2+^ (10 μM) in the presence of NAC (50 μM). Cd^2+^ treatment alone destroyed the orderly arrangements of the three rows of OHCs and a single row of IHCs in the basal, middle, and apical turns. Pretreatment with NAC completely prevented the Cd^2+^-induced destruction of hair cell arrays (Figure 8A). To examine the effect of NAC on apoptosis induced by Cd^2+^ in the organ of Corti, the explants were stained with TRITC (red), DAPI (blue), and TUNEL (green). As shown in Figure 8B, NAC prevented the destruction of hair cell arrays and nuclei, and enhanced the TUNEL-positive cells in the explants. Figure 8C presents the percentage of apoptotic cells in different groups of cells. Cd^2+^-induced apoptosis was significantly prevented by NAC treatment.

### Effect of Cd^2+^ on ERK activation in the cochlea and the protective effect of NAC

**Figure 9:**
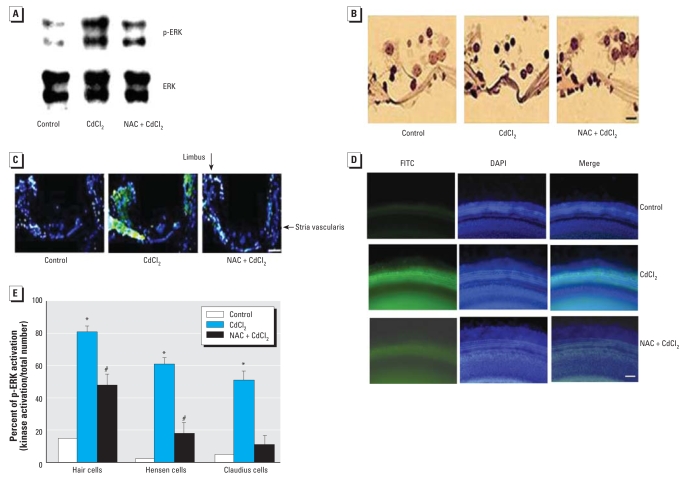
Effect of Cd^2+^ exposure on activation of ERK in mouse cochlea and the protective effect of NAC. (*A*) Western blot analysis showing the effect of Cd^2+^ exposure on p-ERK and the effect of NAC on ERK activation. (*B*) Immunohistochemical staining for p-ERK of outer hair cells and inner hair cells in the organ of Corti. (*C*) Fluorescence micrographs of cochlear sections stained for anti-p-ERK, showing Cd^2+^-induced activation of ERK in the organ of Corti, the stria vascularis, and the limbus in mice cochlea [DAPI (blue) and p-ERK (green)]. (*D*) Immunohistochemical staining for p-ERK (green) and DAPI (blue) in the rat cochlear explants treated with Cd^2+^ (10 μM) for 4 hr showing positive staining of p-ERK; Cd^2+^-activated ERK was blocked with pretreatment with NAC (50 μM for 1 hr). Bars in (*B*) and (*C*) = 10 μm; bar in (*D*) = 50 μm. *p < 0.05 compared with untreated controls. ^#^*p* < 0.05 compared with Cd^2+^ alone.

Mice were exposed to Cd^2+^ through supplied drinking water containing Cd^2+^ (150 mg/L) for 30 days. Some mice also received daily NAC injections during the Cd^2+^ exposure period. After the exposure, the cochleae were removed. One of the two cochleae from each animal was used for Western blot analysis, and one was used for immunohistochemical analysis. Western blot analysis showed that Cd^2+^ exposure induced ERK activation (increased p-ERK level). However, NAC treatment prevented the change (Figure 9A). Immunohistochemical staining showed Cd^2+^-induced ERK activation in the organ of Corti, limbus, and the stria vascularis, but the effects were blocked by NAC treatment (Figure 9B,C) Figure 9D shows immunohistochemical staining of activated ERK (green) in rat cochlear explants exposed to Cd^2+^ alone (10 μM) for 4 hr or Cd^2+^ plus pre-treatment with NAC (50 μM) for 1 hr. Cd^2+^-induced activation of ERK was blocked by NAC pretreatment. The relative p-ERK levels in different cochlear cells (hair cells, Hensen cells, and Claudius cells) are presented in Figure 9E.

### Protective effect of NAC on Cd^2+^-induced ABR threshold shift in mice

**Figure 10:**
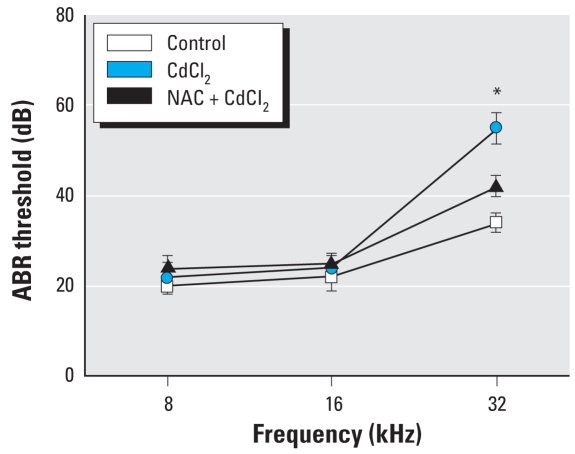
ABR thresholds (mean ± SE) showing effects of Cd^2+^-induced shift at 32 kHz and protective effect of NAC. *p < 0.05 compared with the untreated control.

ABR was recorded at 8, 16, and 32 kHz. Cd^2+^ exposure (150 mg/L in the drinking water for 30 days) caused significant ABR threshold shift at 32 kHz (Figure 10). Daily injections with NAC (50 mg/kg/day) for 30 days prevented the threshold shift (Figure 10).

## Discussion

The high incidence of hearing loss increases in humans who reside in industrialized countries (Abbate et al. 2005; Hodgkinson and Prasher 2006; Hong 2005; Szeszenia-Dabrowska et al. 2004). Irreversible hearing loss is a characteristic effect of a number of heavy metals. Cd^2+^ is a major environmental and occupational hazard because of its widespread use in industry and subsequent release into the environment. The U.S. Environmental Protection Agency has set a limit of 5 ppb Cd^2+^ in drinking water; the U.S. Food and Drug Administration limits the amount of Cd^2+^ in food colors to 15 ppm (FDA 2006); and the U.S. Occupational Safety and Health Administration (OSHA) limits Cd^2+^ in workplace air to 100 μg/m^3^ [Agency for Toxic Substances and Disease Registry (ATSDR) 1999; OSHA 1990, 1992]. Cd^2+^ toxicity has been described as *in vitro* and *in vivo* apoptosis (Lenet et al. 2003; Viaene et al. 2000; Pathak et al. 2006; Pulido et al. 2003), but its molecular mechanism in the auditory system is not fully understood. Our findings in the present study show that the deleterious effect of Cd^2+^ on the organ of Corti and the ototoxicity of Cd^2+^ can be counteracted by antioxidants.

In mammals, mitochondria act as the central checkpoints for many forms of apoptosis. The mitochondrial pathway is believed to be the main target of the survival signaling system (Christophe and Nicolas 2006). The mitochondria commit the cell to undergo apoptosis by *a*) increasing the permeability of the outer mitochondrial membrane and decreasing the mitochondrial transmembrane potential; *b*) releasing cyt c and apoptosis-inducing factor; and *c*) producing ROS. Therefore, in this study we focused on investigating these events. We found that Cd^2+^ induced cell death, MMP loss, ROS generation, and the release of cyt c in auditory HEI-OC1 cells. This suggests that the increased level of ROS production by Cd^2+^ might lead to a decrease in MMP, which in turn increases mitochondrial membrane permeability and the release of mitochondrial apoptogenic factors (cyt c) into the cytosol. However, further study will be needed to clarify the nonmitochondrial signaling pathways and delineate the role of mitochondria-mediated apoptosis in the broader spectrum of the apoptotic signaling mechanisms.

Caspases, a family of cysteine-dependent aspartate-directed proteases, play important roles in initiating and executing apoptosis. Some studies strongly suggest that caspases play a key role in Cd^2+^-induced cell death (Kim et al. 2000; Kondoh et al. 2002; Li et al. 2000). In the present study, Cd^2+^ induced caspase-3, caspase-8, and caspase-9 activation. In addition, caspase-3 inhibitor (Z-DEHD-FMK), and caspase-9 inhibitor (Z-LEHD-FMK) inhibited Cd^2+^-induced cell death, ROS generation, and MMP loss. Therefore, we believe that the apoptosis mechanism of Cd^2+^ in auditory cells might occur, at least in part, through a caspase-dependent pathway. Although Cd^2+^ can induce apoptosis through a caspase-dependent pathway, the effect of Cd^2+^ on the caspase-independent process was not elucidated in this study. Hence, further study will be needed to determine how Cd^2+^ affects the translocation of the apoptosis-inducing factor from the cytosol to the nuclei and how it mediates caspase-independent apoptosis.

MAPK pathways are central components of the intracellular signaling networks that control many aspects of mammalian cellular physiology, including cell proliferation, differentiation, and apoptosis (Wada and Penninger 2004). MAPKs are also likely to be involved in molecular mechanisms for the action of Cd^2+^, as it was reported that the activation of ERK1/2, JNK, and p38 MAPK occurs in renal cells (mesangial or glomerular) (Hirano et al. 2005), macrophages (Misra et al. 2002), and tumor cell lineages (Lee et al. 2005). This discrepancy might be due to differences in cell type. In the present study, we observed that among the three inhibitors, the ERK inhibitor effectively suppressed cell death. From this, we can presuppose that Cd^2+^-induced apoptosis occurs through ERK activation. Therefore, we examined whether Cd^2+^ affects ERK activation *in vitro* and *in vivo*. Cd^2+^ increased the level of ERK activation but had no effect on the activation of p38 and JNK (data not shown). Hence, we hypothesize that Cd^2+^ induces apoptosis in auditory cells by activating ERK.

The cochlear component of hearing is more vulnerable to Cd^2+^ toxicity than other parts of the auditory system (Ozcaglar et al. 2001). In the present study, Cd^2+^ exhibited elevated ABR thresholds at 32 kHz frequency. This result suggested that Cd^2+^ significantly affects the basal turn in mice. Treatment of Cd^2+^ destroyed the orderly arrangements of the three OHC rows and a single row of IHCs in the basal, middle, and apex turns. In addition, we observed that Cd^2+^ induced apoptosis in hair cells, Hensen cells, and Claudius cells in organ of Corti. Hair cells and Hensen cells were more sensitive than Claudius cells to Cd^2+^. This result suggests that Cd^2+^ might affect the mechanical vibration of the basilar membrane and alter neural impulses transmitted to the brain. In addition, we showed that pretreatment with NAC completely prevented destruction of hair cell arrays and apoptosis induced by Cd^2+^.

In our investigation of the *in vivo* effect of NAC against Cd^2+^, we found that the level of ERK activation was increased in the OHCs, limbus, and stria vascularis compared with the control. In animals treated with Cd^2+^ plus NAC, the level of ERK activation was decreased. This suggests that the ERK pathway is a potential therapeutic target for preventing Cd^2+^-induced ototoxic damage. Although NAC attenuated ERK activation, the effect of NAC on the other pathways involving MAPK upstream/downstream and apoptosis marker was not determined. Therefore, further studies will be needed to clarify the role of NAC on the MAPK pathway in the auditory system.

## Conclusion

Cd^2+^ induced cell death, ROS generation, the MMP loss, cyt c release, and caspase-3 and caspase-9 activation, and increased the level of ERK activation in auditory cells. In addition, Cd^2+^ destroyed hair cells in the basal, middle, and apex cochlear turns and induced the activation of ERK. However, in the presence of Cd^2+^, an antioxidant counteracted ototoxicity by suppressing the activation of ERK and caspase and preventing the destruction of the hair cell arrays in primary explants of the rat organ of Corti. These results should improve the understanding of the pharmacologic mechanism and potential treatments for Cd^2+^-induced ototoxicity.
